# Epithelial Ovarian Carcinoma Types and the Coexistence of Ovarian Tumor Conditions

**DOI:** 10.5402/2011/784919

**Published:** 2011-07-14

**Authors:** Catharina. C. van Niekerk, Johan Bulten, José A. A. M. van Dijck, André L. M. Verbeek

**Affiliations:** ^1^Department of Epidemiology, Biostatistics, and HTA, Radboud University Nijmegen Medical Centre, P.O. Box 9101, 6500 HB Nijmegen, The Netherlands; ^2^Department of Pathology, Radboud University Nijmegen Medical Centre, 6500 HB Nijmegen, The Netherlands

## Abstract

*Objective*. Ovarian carcinomas are presumed to arise within ovarian inclusion cysts or from a coexisting epithelial lesion in the ovary. Insight may be gained by relating different subtypes of ovarian cancer with the presence of coexisting tumor-like conditions. *Methods*. The Dutch nation-wide pathology database PALGA (Pathologisch Anatomisch Landelijk Geautomatiseerd Archief) identified the various histopathological subtypes of ovarian cancer in 824 patients diagnosed in 1996–2003, and recorded the presence of epithelial tumor conditions around the ovarian tumors. In addition, a PALGA database of all 153 consecutive patients referred to the Nijmegen University Medical Centre in 2007 for histopathological work-up was analyzed. *Results*. The prevalence of coexisting ovarian tumor conditions was 16.4% (135 out of 824 patients, (95% CI: 8.4%–24.4%)). The coexistence was highest for endometrioid, mucinous, clear cell, and borderline malignancies. The referral group revealed 35% (54 out of 153 patients, (95% CI: 28%–42%)) of coexisting epithelial ovarian tumor conditions. *Conclusion*. One in six patients with a malignant ovarian tumor has a coexisting epithelial tumor condition in the ovary, which is also rather frequently observed in the diagnostic work-up practice.

## 1. Introduction

In developed countries, ovarian cancer is a commonly occurring neoplasm, ranking the 7th and 6th most frequent position for incidence and mortality, respectively [1]. High-incidence areas are Europe and North America, making it an important public health issue [2].

Epithelial ovarian tumors are thought to arise from the surface epithelium (mesothelium) of the ovary [3]. Most early-stage ovarian cancer produce no symptoms, and therefore the majority of the patients present with advanced disease, making prognosis poor. So far, pathologists have devoted very little attention to early ovarian cancer originating in a coexisting benign epithelial lesion [4]. The precise origin of this epithelium is controversial; one hypothesis argues that the mesothelium lining of the ovarian surface undergoes a Müllerian metaplasia; another one that the same epithelium is derived from the fallopian tube or uterus via passive transport [5].

There are four main types of epithelial ovarian cancer: serous ovarian carcinoma (30% to 70%), mucinous ovarian carcinoma (5% to 20%), endometrioid ovarian carcinoma (10% to 20%), clear cell carcinoma (3% to 10%), and furthermore a minor group of transitional cell or Brenner tumor (1%), and mixed epithelial carcinoma (3%) [6–8]. As described by Mok et al. [9] the progression model of epithelial ovarian cancer is complicated, when the different histological subtypes of ovarian neoplasms are considered. One theory is that ovarian carcinoma arises from endometriosis, the ectopic occurrence of endometrial tissue [10]. Especially clear cell and endometrioid carcinomas are thought to arise from foci of endometriosis [11]. Associations between endometriosis and ovarian carcinoma have been found, but could also be explained by shared risk factors for the coexistence of the conditions [11]. 

The ovarian surface epithelium (OSE) is generally regarded as the precursor of epithelial ovarian tumors. Recent evidence, however, suggests that the fallopian tube could also be the source of some subtypes of epithelial ovarian cancers [12]. Since the ovaries and tubes are closely related, it is hypothesized that cells of the tubes can mimic ovarian cancer [13, 14]. 

The aim of this study is to investigate the coexistence of different epithelial subtypes of ovarian carcinoma, with tumor-like conditions [15], benign tumors, and borderline malignancies, in relation to age under and above 55 years at diagnosis, as an approach to distinguish the development of pre- and postmenopausal pathways.

## 2. Materials and Methods

We used a random sample (1000 patients out of 9000 patients) out of the database of the nation-wide pathology database PALGA in the Netherlands [16] of the years 1996–2003, from which 824 patients appeared to be diagnosed with an epithelial ovarian cancer. Records in the PALGA database contain comprehensive summaries of the pathology reports and diagnostic codes similar to the systematized nomenclature of medicine (SNOMED) classification of the College of American Pathologists [16]. We scrutinized all reports of 20 lines on average, (>250 words) of the random sample of 1000 patients with an ovary-related diagnosis. Of these 824 patients, with an epithelial ovarian malignancy we collected all other records from PALGA linked with histopathological confirmed diagnoses of borderline malignancies, benign tumors, and tumor-like conditions (endometriosis, simple cysts, endometriotic cysts, and inclusion cysts (two ruptured cysts and one cyst wall)) in the ovary. In case of a diagnose of borderline malignancy as well as malignant carcinoma, in the same patient, the borderline tumor was seen as a coexisting condition.

Considering the impact of the findings to current medical practice, we also studied all PALGA receipts of the PALGA database of the pathology department at the Radboud University Nijmegen Medical Centre. It contained the records of 153 patients diagnosed with an ovary-related diagnosis. The Scientific Committee of PALGA approved the study protocol.

### 2.1. Data Analyses

Descriptive analysis was applied to learn from the association between histopathologically subtypes of ovarian epithelial cancer and benign epithelial neoplasms and tumor-like conditions. Anticipating different developmental pre/postmenopausal pathways from benign to malignant tumors, age at diagnosis was dichotomized at 55 year. The Chi-square test was used to arrive at *P*-values, and 95% confidence intervals for proportions were calculated.

## 3. Results

We studied 824 patients with ovarian tumor; 806 had malignant carcinomas and 18 had borderline lesions without carcinoma lesions. In 135 women (16.4%) coexisting ovarian cancer lesions were observed (95% CI: 8.4%–24.4%). In 21 patients two different types of coexisting ovarian tumor lesions or tumor-like conditions were noticed. Of the 806 patients with malignant ovarian cancer, 129 patients (16.0%) had also a tumor-like condition (*n* = 38), a benign tumor (*n* = 47), or a borderline malignancy (*n* = 44). Of the 18 patients with borderline malignancies, 6 (33.0%) had coexisting lesions. The presence of possible precursors according to the different histological subtypes and for both age categories is shown in Table 1. Coexisting epithelial tumor conditions were more prevalent in the age category <55 years. Overall, the ratio of the prevalences of the under age 55 versus 55+ group was 1.71 (95% CI: 1.26–2.31). In particular the subtypes of endometrioid, mucinous, clear cell, and borderline malignancies showed excess prevalences for women under age 55 years.

Of the 153 consecutive patients from the Department of Pathology of the UMCN in 2007, 54 out of 153 patients did not have coexisting ovarian tumor conditions or abnormalities. A number of 22 patients had nonepithelial lesions while for two lesions it could not be established whether the tumors were primarily linked to the ovaries or were metastases from elsewhere. The remaining 75 records concerned epithelial ovarian lesions ranging from tumor-like conditions (inclusion cysts and simple cysts *n* = 29), adenomas (*n* = 15), borderline malignancies (*n* = 6), to malignant tumors (*n* = 25); see Table 2. One-third were actually cancer, mainly serous carcinomas and adenocarcinomas not otherwise specified, of which 4 patients (16.0%) also showed coexisting epithelial tumor lesions or tumor-like conditions. This means that the hospital referral group revealed 35% (54 out of 153 patients) of coexisting epithelial ovarian tumor conditions.

Of the lesions considered as coexisting epithelial tumor conditions, 58.0% (29/50) were tumor-like conditions, 40.0% (15/50) adenomas, and 12.0% (6/50) borderline malignancies. Of the carcinoma patients (25), 32.0% were younger than 55 years old while of the borderline malignancy patients 67.0% (6/10) were younger than 55. Of patients with adenomas or tumor-like conditions, 80.0% and 82.8% were under age 55 (Table 2).

Coexisting conditions were almost similar to the principle ovary cancer in case of borderline malignancies (83%) and less similar in the other types of malignancies (46–64%).

## 4. Discussion

### 4.1. The PALGA Database 1996–2003

One out of six patients with a malignant epithelial ovarian cancer did have a coexisting ovarian benign lesion. Tumor-like conditions and adenomas were the most commonly observed benign lesions whereas serous, endometrioid, and mucinous carcinomas were the most common malignant tumors. Tumor-like conditions, especially in endometrioid carcinomas, were very frequent (data not shown). One in three of these cases was an endometrioid cyst or endometriosis. The actual number of endometrioid cysts may have been even larger, indicating a relation between endometriosis, endometrioid cyst and endometrioid carcinoma [9, 11, 17]. Nevertheless, because of the cross-sectional nature of our study, causality cannot be deferred, which means that this relation may also be explained by shared risk factors [11].

Ovarian cancer patients who had a malignant as well as a benign lesion were generally younger than women with only a malignant tumor. This may underpin the fact that benign tumors tend to occur at younger age than malignant tumors. 

As stated earlier, only part of the malignant epithelial ovarian tumors will arise from a coexisting benign epithelial lesion [4, 18]. As endometrioid, mucinous, clear cell, and borderline malignant epithelial tumors are more common in women with a benign and a malignant lesion, these subtypes are the most likely to develop through tumor transition. Recently it is hypothesized that most high-grade serous carcinomas (50% or more) arise from the distal fallopian tube (fimbria) [19]. Our results may not be in conflict with this hypothesis while only a small number (10.9%) of serous carcinomas had coexisting lesions. A subset will develop *de novo* from the ovarian surface epithelium or its inclusion cysts [20]. 

As described by Auersperg et al. [21] the surface epithelium of the ovary and tubal epithelium were considered as a unit, with an area of increased susceptibility to neoplastic progression encompassing the ovarian surface epithelium and the distal fimbriae. Progression along different histotypic pathways might then be influenced by microenvironmental factors and mutational events.

### 4.2. Findings of the PALGA Nijmegen 2007 Database

Women with a tumor-like condition or adenoma appeared to be younger (82.8% at age <55 year and 80.0%, resp.) than women with a malignant tumor (32.0% at age <55 years). The majority of patients with a borderline malignancy also were younger than 55 years (66.6%). 

Describing the occurrence of benign ovarian tumors in itself is also very difficult. As stated earlier, malignant ovarian tumors are often diagnosed in a late stage; benign tumors are often not detected at all, for example, when an obvious carcinoma is present within the ovary, most pathologists would not mention the presence of cortical inclusion cysts. Therefore, in daily gynecological practice most of the benign tumors may be detected coincidentally. This, however, only seems to be the tip of the iceberg: most benign tumors remain undetected. Consequently, it is hard to study at which age what kind of benign tumors occur.

A limitation of the PALGA Nijmegen 2007 database was that it was unclear whether the patients without abnormality or benign lesion were representing the general population. Some of the patients had the ovaries removed because they were carriers of a mutation in BRCA1 or BRCA2, which sets them at high risk to develop ovarian cancer. Other patients were examined because they had symptoms. Due to this fact, the incidence of benign lesions is probably higher than in the general population. It is unknown whether the larger dataset also contains BRCA/high-risk women as well.

## 5. Conclusion

The strength of this study is the analysis specified to the various histological subtypes in relation to possible precursors of ovarian cancer and malignancies. Overall, the prevalence of coexisting epithelial ovarian tumor conditions was 1.71-times (95% CI: 1.26–2.31) as high in patients <55 years versus patients ≥55 years. Unfortunately, sparse numbers of cases hamper to arrive at a meaningful conclusion on more aggressive malignancies in younger women. The histological subtypes endometrioid, mucinous, clear cell, and borderline malignancies showed the highest prevalences of coexisting lesions. 

Weaknesses of the study lie in its retrospective design and the fact that coexisting pathologies are usually overlooked or better said underreported. Further research is needed to confirm the results in larger study populations, and possibly be combined with a study of (pre-)neoplasia arising in the Fallopian tube in relation to the hereditary serous ovarian carcinogenesis hypothesis from Piek [13].

The analysis of the PALGA Nijmegen 2007 database indicates that half of all patients referred to the University Medical Centre St. Radboud with tissue specimen sent to the pathology department, have epithelial ovarian tumor conditions. Two-thirds of the latter seem to have coexisting epithelial tumor conditions, adding to the progression to the malignant disease stage. These findings may prompt to not only reporting ovary cancer but also coexisting ovarian tumor conditions. The latter if detected in the absence of ovary cancer need follow-up surveillance.

##  Funding

No funding was received for this paper.

## Figures and Tables

**Table 1:** The occurrence of coexisting ovarian tumor conditions in epithelial ovarian cancer according to subtypes and the age at diagnosis in 824 patients.

Ovarian cancer subtype and age category	Total	1 or 2 lesions present*		Ratio of prevalences (95% CI)
	*n*	*n*	%*	
All subtypes	824	135	16.4	
<55 yr	274	62	22.6	1.71 (1.26–2.31)
≥55 yr	550	73	13.3	1
Serous carcinoma	385	42	10.9	
<55 yr	113	15	13.3	1.33 (0.74–2.42)
≥55 yr	272	27	9.9	1
Endometrioid carcinoma	113	25	22.1	
<55 yr	46	13	28.3	1.58 (0.79–3.14)
≥55 yr	67	12	17.9	1
Mucinous carcinoma	98	28	28.6	
<55 yr	47	16	34.0	1.45 (0.76–2.73)
≥55 yr	51	12	23.5	1
Adenocarcinoma	73	12	16.4	
<55 yr	17	3	17.6	1.10 (0.33–3.60)
≥55 yr	56	9	16.1	1
Clear cell carcinoma	56	11	19.6	
<55 yr	23	7	30.4	2.51 (0.83–7.60)
≥55 yr	33	4	12.1	1
Mixed malignancy	34	5	14.7	
<55 yr	11	2	18.2	1.39 (0.27–7.18)
≥55 yr	23	3	13.0	1
Borderline malignancy	18	6	33.3	
<55 yr	11	4	36.4	1.27 (0.31–5.20)
≥55 yr	7	2	28.6	1
Other	47	6	12.8	
<55 yr	6	2	33.3	3.41 (0.79–14.80)
≥55 yr	41	4	9.8	1

*Lesions present: tumor-like condition (endometriosis, simple cysts, endometriotic cysts, inclusion cysts), benign tumor, and borderline malignancy.

95% CI, confidence interval.

**Table 2:** Specific diagnoses of 75 consecutive tissue specimen from PALGA Nijmegen 2007 according to age.

Diagnosis	All ages	<55 yr		≥55 yr	
	*n*	*n*	%	*n*	%
Total	75	48	64.0	27	36.0
Ovarian carcinoma	25*	8	32.0	17	68.0
Borderline malignancies	6	4	66.6	2	33.3
Adenoma	15	12	80.0	3	20.0
Tumor-like conditions**	29	24	82.8	5	17.2

*4 patients (16.0%) showed coexisting epithelial ovarian tumor conditions.

**tumor-like conditions: simple cysts, endometriotic cysts, inclusion cysts.

Coexisting epithelial ovarian carcinoma conditions: (4 + 6 + 15 + 29)/75 = 72%.

Chi-square = 17.225, df = 3, *P*-value = 0.001.
